# The effect of the D614G substitution on the structure of the spike glycoprotein of SARS-CoV-2

**DOI:** 10.1073/pnas.2022586118

**Published:** 2021-02-12

**Authors:** Donald J. Benton, Antoni G. Wrobel, Chloë Roustan, Annabel Borg, Pengqi Xu, Stephen R. Martin, Peter B. Rosenthal, John J. Skehel, Steven J. Gamblin

**Affiliations:** ^a^Structural Biology of Disease Processes Laboratory, Francis Crick Institute, London NW1 1AT, United Kingdom;; ^b^Structural Biology Science Technology Platform, Francis Crick Institute, London NW1 1AT, United Kingdom;; ^c^Precision Medicine Center, The Seventh Affiliated Hospital, Sun Yat-sen University, 518107 Shenzhen, Guangdong, China;; ^d^Structural Biology of Cells and Viruses Laboratory, Francis Crick Institute, London NW1 1AT, United Kingdom

**Keywords:** SARS-CoV-2, Coronavirus, spike, cryo-EM, D614G

## Abstract

The majority of currently circulating severe acute respiratory syndrome coronavirus 2 (SARS-CoV-2) viruses have mutant spike glycoproteins that contain the D614G substitution. Several studies have suggested that spikes with this substitution are associated with higher virus infectivity. We use cryo-electron microscopy to compare G614 and D614 spikes and show that the G614 mutant spike adopts a range of more open conformations that may facilitate binding to the SARS-CoV-2 receptor, ACE2, and the subsequent structural rearrangements required for viral membrane fusion.

## Significance

The spike proteins of most current severe acute respiratory syndrome coronavirus 2 isolates contain a D614G substitution, by comparison with the spike protein of initial isolates. In this study we present high-resolution, single-particle cryo-electron microscopy structures of the G614 spike variant showing that it adopts a predominantly open conformation, unlike the D614 spike that is mostly closed. We conclude that the D614G substitution promotes “opening” of the spike, priming it for binding to the receptor ACE2 and possibly for its subsequent role in membrane fusion. The observed open conformation of the G614 spike may be the reason for the current virus’ reported increased infectivity and its current predominance.

The spike glycoproteins of coronaviruses are responsible for receptor binding and membrane fusion during the initial stages of virus infection (1). Viruses that have spike proteins containing the amino acid substitution D614G are currently predominant in the severe acute respiratory syndrome coronavirus 2 (SARS-CoV-2) pandemic, and it has recently been shown that G614 viruses have higher infectivity and produce higher viral loads than D614 viruses (2–4).

We, and others, have shown that the D614 spike adopts several different conformations, including a “closed” conformation in which the receptor-binding domain (RBD) is partly buried and cannot bind to the human ACE2 receptor. We have also shown that both furin cleavage (5) and the presence of ACE2 (6) increase the proportion of the spikes that adopt open conformations and suggested that the D614G substitution could also promote the spike’s “opening” (6). To better understand the impact of the D614G substitution we have now solved the cryo-electron microscopy (cryo-EM) structure of the G614 spike and compared it to that of the D614 spike recently solved by us and others (5, 7, 8).

## Results

**Fig. 1.**
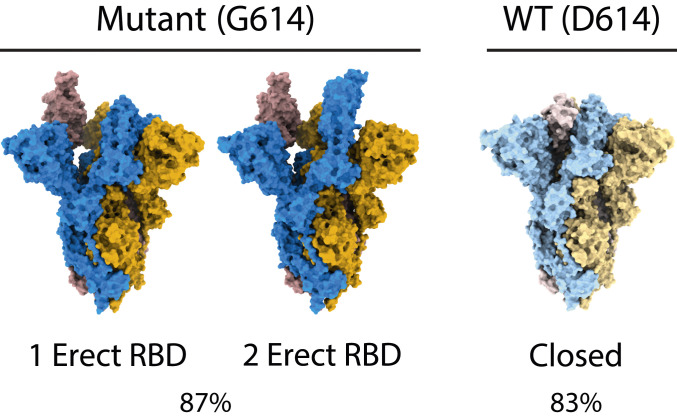
Space-filling representation of the two predominant open forms of G614 spike and the predominant closed form of D614 spike. The one- and two-RBD-erect forms of G614 spike make up 87% of particles, while the closed form represents 83% of the D614 spike particles in our previous study (5). The three subunits of spike are colored in blue, goldenrod, and rosy brown for G614 and in lighter shades of the same colors for D614. The molecule is viewed with its long axis vertical.

**Fig. 2.**
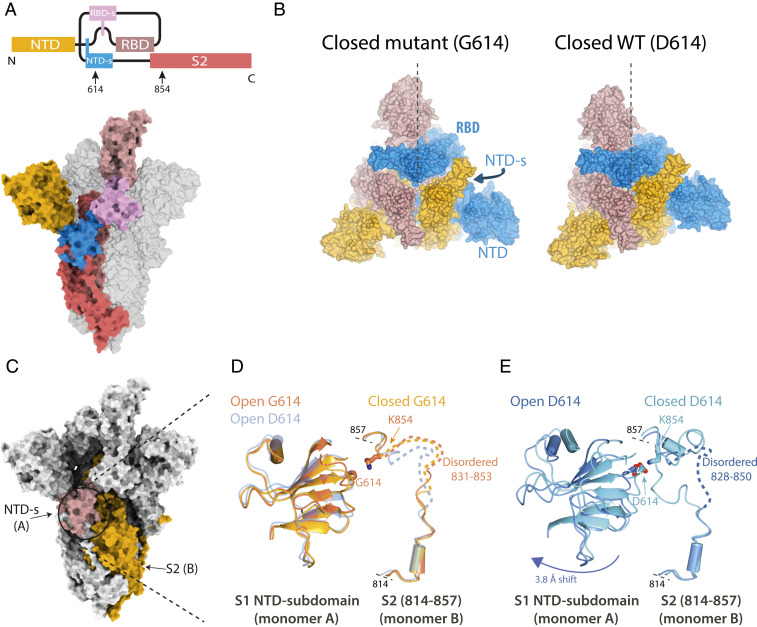
Structural differences between the G614 and D614 spike. (*A*) Bar diagram, showing domain connectivity of spike and locations of residues 614 and 854 (*Top*), with domains colored on single monomer of open spike trimer and other monomers shown in gray (*Bottom*). (*B*) Space-filling representation of the closed conformations of G614 and D614 spikes viewed down the threefold axis. The monomers are colored similarly to Fig. 1 and the positions of the NTD, NTD-associated subdomain (NTD-s), and the RBD are labeled for one monomer, with the vertical line emanating from the center of the trimer helping to distinguish the different conformations of the NTDs in the two structures; the trimers are aligned on the large S2 helix (residues 986 to 1032) of one monomer. (*C*–*E*) Interaction between S1 NTD-s of the erecting monomer with a segment of S2 domain from a neighboring chain. The overall spike structure is shown as a molecular surface with the two interacting domains, NTD-s and S2, from two adjacent monomers shown in rosy brown and yellow (*C*), with (*D*) a zoom of the NTD-s in cartoon representation with the two residues forming a salt bridge in the closed conformation in ball-and-stick representation. *D* also shows an overlay of the similar structures of the open (colored in gold) and closed (colored orange) forms of G614 with the open form of D614 (colored light blue). (*E*) An overlay of the distinct structures of the open (dark blue) and closed form (light blue) of D614.

The expressed G614 protein was stable and suitable for high-resolution single-particle cryo-EM reconstruction. Strikingly, the structures we solved (Table 1) show that the G614 spike exists predominantly in the open, receptor-binding-competent conformation, in contrast to the D614 spike (Figs. 1 and 2*A*); 87% of G614 spikes have either one or two erect RBDs, compared with 17% in our D614 structure (5) [and less than 50% in the structures of D614 reported elsewhere (7, 8), none of which showed two erect RBDs]. G614 spikes with two erect RBDs account for 20% of observed particles. This conformation has only previously been observed for the furin-cleaved protein on exposure to ACE2 (6), including by tomography on virions (9).

**Table 1.** Cryo-EM data collection, refinement, and validation statistics

	Closed D614G (EMD-12229) (PDB ID code 7BNM)	One erect RBD D614G (EMD-12230) (PDB ID code 7BNN)	Two erect RBDs D614G (EMD-12231) (PDB ID code 7BNO)
Data collection and processing			
Voltage, kV	300	300	300
Electron exposure, e^–^/Å^2^	36.8	36.8	36.8
Defocus range, μm	−1.0 to -3.0	−1.0 to -3.0	−1.0 to -3.0
Pixel size, Å	0.85	0.85	0.85
Symmetry imposed	C3	C1	C1
No. of final particle images	30,000	150,000	45,000
Map resolution, Å (FSC threshold = 0.143)	3.6	3.5	4.2
Map resolution range, Å	3.5–5.5	3.5–5.5	4.0–8.0
Refinement			
Initial model used (PDB ID code)	6ZGE	6ZGG	7A93
Model resolution, Å (FSC threshold = 0.5)	3.7	3.6	4.4
Map sharpening *B* factor, Å^2^	−102.7	−115.3	−96.7
Model composition			
Nonhydrogen atoms	25,971	25,788	25,509
Protein residues	3,207	3,207	3,197
Ligands	63	50	34
Rmsd			
Bond lengths, Å	0.008	0.003	0.007
Bond angles, °	0.787	0.576	0.989
Validation			
MolProbity score	1.88	2.11	2.15
Clashscore	9.22	11.10	12.94
Poor rotamers, %	0.54	0.00	0.75
Ramachandran plot			
Favored, %	94.26	93.05	90.67
Allowed, %	5.74	6.95	9.33
Disallowed, %	0.00	0.00	0.00

The minority of G614 spikes (13%) that do not have one or more RBDs erect adopt a partially closed conformation, which is less tightly packed than the closed conformation of the D614 spike, with several constituents of the G614 spike displaying more local flexibility. Comparison of the contact area between each monomer of the trimer with a single neighboring monomer indicates 4,000 Å^2^ for the closed conformation of the G614 spikes, compared to 5,900 Å^2^ for the closed D614 spike (5). This substantial change in contact area is clearly evident in Fig. 2*B*, where the RBDs are not packed as closely together in the G614 spikes as they are in the D614 spikes. This more dilated packing arrangement in G614 spikes is also accompanied by a clockwise, rigid-body rotation of the N-terminal domains (NTDs) (as viewed in Fig. 2*B*) by comparison with the D614 spikes (a similar direction of movement to that observed for D614 spikes upon transition from the closed to the open conformation). There are several regions at the virus-membrane-distal top of the G614 spikes in this closed conformation that display more local flexibility, associated with poorer local resolution (*SI Appendix*, Fig. 1), than the corresponding regions of the D614 spikes, presumably resulting from the substantial decrease in monomer packing surface. Most notably, these regions include the large loop (residues 468 to 491) at the tip of the RBD and to a lesser extent the rest of the surface of the receptor-binding motif of the RBD as well as surface loops of the NTD that are furthest away from the symmetry axis. Consistent with this looser packing of the RBDs in the closed form of the G614 spike is the lack of density for a bound fatty acid (10) that is present in our D614 spikes close to the RBD/RBD binding interface (5).

Inspection of the closed and open G614 and D614 spike structures reveals the likely mechanistic reason for the differences in domain orientation (Fig. 2 *C*–*E*). As reported before, the residue D614, located on the NTD-associated subdomain, in the closed conformation of the D614 spike forms a salt bridge with K854 from the S2 component of the neighboring monomer (5). Concomitant with opening of the RBD, and subsequent receptor engagement, the NTD-associated subdomain undergoes structural rearrangements and a rigid-body shift that lead to breaking of the D614–K854 salt bridge (Fig. 2*D*). Disruption of the salt bridge correlates with unfolding of the 827 to 855 region of S2, which may prime the downstream, putative fusion-peptide-containing sequence (residues 815 to 825) for membrane fusion, as the S1 and S2 chains become more separate (6). With a glycine residue at position 614 a salt bridge cannot be formed and thus the structures of the G614 spike in the open and closed conformation are very similar to each other in this region and, importantly, similar to the open conformation of the D614 spike **(**Fig. 2*D*). In contrast, the local structures of the D614 spike in this region are markedly different between the open and closed forms (Fig. 2*E*).

## Discussion

A number of biological features have been reported to distinguish the D614G mutant that may correlate with these differences in structure. They include in particular higher virus loads based on estimates of virus RNA in the infected respiratory tract (4), increases in case fatality (11), and up to ninefold increases in virus production in vitro (2, 12). There does not appear to be evidence of increased transmissibility (13), but from genetic analysis increases in distribution of the variant are consistent with a selective advantage (14). Recently, Yurkovetskiy et al. have addressed several aspects of the mutant viruses’ properties, including the structural basis of the closed/open conformation of the spike, that raise two issues pertinent to our work (12). First, conclusions are made about the extent of the open RBD conformations of G614 spikes that are not corroborated by deposited models or density maps. Our analysis of the deposited density map (12) indicates that it is highly anisotropic, restricting reliable interpretation of the RBD location (*SI Appendix*, Fig. 2 *A* and *B*). Second, Yurkovetskiy et al. (12) suggest that the effect of the D614G substitution is mediated by the loss of a hydrogen bond to a threonine residue at position 859 of S2. This mechanism is inferred from earlier studies (4, 7) that did not allow unambiguous modeling of the protein structure in the described region. Considering earlier work from our group and others (6, 9, 15) on the closed RBD form of spike (*SI Appendix*, Fig. 2*C*), our conclusion is that the evidence strongly supports the role of a salt bridge between D614 and K854, and not T859, in stabilizing the closed spike conformation.

As a consequence of the D614G substitution, a tightly closed structure observed for the D614 spike is not formed and the G614 spike assembly adopts a greater range of more open and flexible conformations than its D614 counterpart. The greater conformational flexibility of the G614 spike explains how it more readily adopts the receptor-binding-competent conformation and suggests how the presently circulating strains of SARS-CoV-2 might have fitness advantage over the D614 variant and display higher infectivity. At the same time, the more open conformation may result in the exposure of epitopes for additional neutralizing antibodies; indeed, recent reports suggest that the D614G substitution increases the susceptibility of SARS-CoV-2 to neutralization (16). As a consequence, as populations become more exposed to SARS-CoV-2, and as more people acquire an immune reaction to the virus, it is likely that the G614 form will have less selective advantage.

## Materials and Methods

### Protein Production.

The construct coding for the D614G mutant was based on the furin-uncleavable version of the SARS-CoV-2 spike protein ectodomain with a set of stabilizing mutations (R682S, R685S, K986P, and K987P) that we described before (5).

The G614 spike protein was produced very similarly to the D614 spike we described before (5). Briefly, it was expressed in Expi293F cells (Gibco) growing in suspension at 37 °C in an 8% CO_2_ atmosphere transfected with ExpiFectamine 293 (Gibco) and 1 mg of DNA per liter of culture. The enhancers were added 20 h after the transfection, according to the manufacturer’s instructions (Gibco), and the cells were then moved to 32 °C and the supernatant containing the protein was harvested on the fifth day posttransfection. The collected supernatant was clarified, bound overnight to TALON beads (Takara), briefly washed, eluted with 200 mM imidazole, concentrated, and gel-filtered on a Superdex 200 Increase 10/300 GL column (GE Life Sciences) into 150 mM NaCl and 20 mM Tris, pH 8.

### Cryo-EM Sample Preparation and Data Collection.

The G614 spike glycoprotein was frozen on 200-mesh Quantifoil R2/2 grids glow-discharged for 30 s at 25 mA. Four microliters of spike protein at ∼0.5 mg/mL in 150 mM NaCl and 20 mM Tris, pH 8, supplemented with 0.1% octyl glucoside was applied on a grid at 4 °C, blotted for 4 to 4.5 s using a Vitrobot MkIII, and plunge-frozen into liquid ethane.

Data were collected using a Titan Krios operating at 300 kV. Images were recorded using a Falcon III detector operating in electron counting mode. Images were recorded as a 40-s exposure, fractionated into 32 frames, with an accumulated dose of 36.8 e/Å^2^. The calibrated pixel size was 0.85 Å and images were collected at various defoci between 1.0 and 3.0 µm.

### Cryo-EM Data Processing.

The frames of the collected movies were aligned using MotionCor2 (17) implemented in RELION (18), and the Contrast Transfer Function (CTF) was fitted using CTFfind4 (19). Particles were picked using crYOLO (20) using a model trained on manually picked micrographs. Picked particles were subjected to two rounds of two-dimensional classification in cryoSPARC (21), retaining classes with clear secondary structure. An ab initio three-dimensional (3D) model was generated using cryoSPARC, which was used as an initial model for 3D classification in RELION, separating into 10 classes. Particles in classes which pertained to the closed form, one erect RBD form, and two erect RBDs form were refined using RELION 3D-Autorefine, followed by Bayesian polishing (22). The final refinements were carried out in cryoSPARC using the homogeneous refinement protocol, coupled to CTF refinement. C3 symmetry was imposed on the closed form. The final maps had local resolution estimated using blocres (23) implemented in cryoSPARC, followed by local resolution filtering and global B-factor sharpening (24) in cryoSPARC. The image processing workflow is summarized in *SI Appendix*, Fig. 3.

### Model Building.

Models were built based on our previously published structures for the closed wild-type SARS-CoV-2 spike (Protein Data Bank [PDB] ID code 6ZGE) (5), one erect RBD (PDB ID code 6ZGG) (5), and two erect RBDs (PDB ID code 7A93) (6). Models were fitted into the density and manually adjusted using Coot (25). The closed structure had an S1 structure with large deviations in the positioning of the S1 subdomains. The model was initially built by rigid body refinement in PHENIX (26), followed by adjustment in Coot. All models were real-space-refined and validated using PHENIX (Table 1). Accuracy of model building for previously deposited D614 structures was compared by measuring Q-scores using the UCSF Chimera plugin (27, 28).

## Supplementary Material

Supplementary File

## Data Availability

Maps and models have been deposited in the Electron Microscopy Data Bank, https://www.ebi.ac.uk/pdbe/emdb/ (accession nos. EMD-12229, EMD-12230, and EMD-12231). Models have been deposited in the Protein Data Bank, https://www.ebi.ac.uk/pdbe/ (PDB ID codes 7BNM, 7BNN, and 7BNO).

## Change History

August 23, 2021: The text of this article has been updated; please see accompanying Correction for details.

## References

[1] F. Li, Structure, function, and evolution of coronavirus spike proteins. Annu. Rev. Virol. 3, 237–261 (2016). 27578435 10.1146/annurev-virology-110615-042301 PMC5457962

[2] L. Zhang., SARS-CoV-2 spike-protein D614G mutation increases virion spike density and infectivity. Nat Commun 11 (1), 6013 (2020). 33243994 10.1038/s41467-020-19808-4 PMC7693302

[3] J. Hu., The D614G mutation of SARS-CoV-2 spike protein enhances viral infectivity and decreases neutralization sensitivity to individual convalescent sera. bioRxiv [Preprint] (2020). https://www.biorxiv.org/content/10.1101/2020.06.20.161323v1.article-info. Accessed 29 October 2020.

[4] B. Korber.; Sheffield COVID-19 Genomics Group, Tracking changes in SARS-CoV-2 spike: Evidence that D614G increases infectivity of the COVID-19 virus. Cell 182, 812–827.e19 (2020). 32697968 10.1016/j.cell.2020.06.043 PMC7332439

[5] A. G. Wrobel., SARS-CoV-2 and bat RaTG13 spike glycoprotein structures inform on virus evolution and furin-cleavage effects. Nat. Struct. Mol. Biol. 27, 763–767 (2020). 32647346 10.1038/s41594-020-0468-7 PMC7610980

[6] D. J. Benton., Receptor binding and priming of the spike protein of SARS-CoV-2 for membrane fusion. Nature 588, 327–330 (2020). 32942285 10.1038/s41586-020-2772-0 PMC7116727

[7] D. Wrapp., Cryo-EM structure of the 2019-nCoV spike in the prefusion conformation. Science 367, 1260–1263 (2020). 32075877 10.1126/science.abb2507 PMC7164637

[8] A. C. Walls., Structure, function, and antigenicity of the SARS-CoV-2 spike glycoprotein. Cell 181, 281–292.e6 (2020). 32155444 10.1016/j.cell.2020.02.058 PMC7102599

[9] Z. Ke., Structures and distributions of SARS-CoV-2 spike proteins on intact virions. Nature 588, 498–502 (2020). 32805734 10.1038/s41586-020-2665-2 PMC7116492

[10] C. Toelzer., Free fatty acid binding pocket in the locked structure of SARS-CoV-2 spike protein. Science 370, 725–730 (2020). 32958580 10.1126/science.abd3255 PMC8050947

[11] M. Becerra-Flores, T. Cardozo, SARS-CoV-2 viral spike G614 mutation exhibits higher case fatality rate. Int. J. Clin. Pract. 74, e13525 (2020). 32374903 10.1111/ijcp.13525 PMC7267315

[12] L. Yurkovetskiy., Structural and functional analysis of the D614G SARS-CoV-2 spike protein variant. Cell 183, 739–751.e8 (2020). 32991842 10.1016/j.cell.2020.09.032 PMC7492024

[13] L. van Dorp., No evidence for increased transmissibility from recurrent mutations in SARS-CoV-2. Nat. Commun. 11, 5986 (2020). 33239633 10.1038/s41467-020-19818-2 PMC7688939

[14] E. M. Volz., Evaluating the effects of SARS-CoV-2 Spike mutation D614G on transmissibility and pathogenicity. Cell 184, 64–75.e11 (2020). 33275900 10.1016/j.cell.2020.11.020 PMC7674007

[15] X. Xiong.; CITIID-NIHR COVID-19 BioResource Collaboration, A thermostable, closed SARS-CoV-2 spike protein trimer. Nat. Struct. Mol. Biol. 27, 934–941 (2020). 32737467 10.1038/s41594-020-0478-5 PMC7116388

[16] D. Weissman., D614G spike mutation increases SARS CoV-2 susceptibility to neutralization. Cell Host Microbe 29, 23–31.e4 (2020). 33306985 10.1016/j.chom.2020.11.012 PMC7707640

[17] S. Q. Zheng., MotionCor2: Anisotropic correction of beam-induced motion for improved cryo-electron microscopy. Nat. Methods 14, 331–332 (2017). 28250466 10.1038/nmeth.4193 PMC5494038

[18] S. H. W. Scheres, RELION: Implementation of a Bayesian approach to cryo-EM structure determination. J. Struct. Biol. 180, 519–530 (2012). 23000701 10.1016/j.jsb.2012.09.006 PMC3690530

[19] A. Rohou, N. Grigorieff, CTFFIND4: Fast and accurate defocus estimation from electron micrographs. J. Struct. Biol. 192, 216–221 (2015). 26278980 10.1016/j.jsb.2015.08.008 PMC6760662

[20] T. Wagner., SPHIRE-crYOLO is a fast and accurate fully automated particle picker for cryo-EM. Commun. Biol. 2, 218 (2019). 31240256 10.1038/s42003-019-0437-z PMC6584505

[21] A. Punjani, J. L. Rubinstein, D. J. Fleet, M. A. Brubaker, cryoSPARC: Algorithms for rapid unsupervised cryo-EM structure determination. Nat. Methods 14, 290–296 (2017). 28165473 10.1038/nmeth.4169

[22] J. Zivanov, T. Nakane, S. H. W. Scheres, A Bayesian approach to beam-induced motion correction in cryo-EM single-particle analysis. IUCrJ 6, 5–17 (2019). 10.1107/S205225251801463X PMC6327179 30713699

[23] G. Cardone, J. B. Heymann, A. C. Steven, One number does not fit all: Mapping local variations in resolution in cryo-EM reconstructions. J. Struct. Biol. 184, 226–236 (2013). 23954653 10.1016/j.jsb.2013.08.002 PMC3837392

[24] P. B. Rosenthal, R. Henderson, Optimal determination of particle orientation, absolute hand, and contrast loss in single-particle electron cryomicroscopy. J. Mol. Biol. 333, 721–745 (2003). 14568533 10.1016/j.jmb.2003.07.013

[25] P. Emsley, B. Lohkamp, W. G. Scott, K. Cowtan, Features and development of Coot. Acta Crystallogr. D Biol. Crystallogr. 66, 486–501 (2010). 20383002 10.1107/S0907444910007493 PMC2852313

[26] P. D. Adams., PHENIX: A comprehensive Python-based system for macromolecular structure solution. Acta Crystallogr. D Biol. Crystallogr. 66, 213–221 (2010). 20124702 10.1107/S0907444909052925 PMC2815670

[27] E. F. Pettersen., UCSF Chimera–A visualization system for exploratory research and analysis. J. Comput. Chem. 25, 1605–1612 (2004). 15264254 10.1002/jcc.20084

[28] G. Pintilie., Measurement of atom resolvability in cryo-EM maps with Q-scores. Nat. Methods 17, 328–334 (2020). 32042190 10.1038/s41592-020-0731-1 PMC7446556

