# Supplementary material for: The effect of the D614G substitution on the structure of the spike glycoprotein of SARS-CoV-2
Source: Proc Natl Acad Sci U S A. 2021 Feb 12;118(9):e2022586118. doi: 10.1073/pnas.2022586118 (PMC7936381; doi:10.1073/pnas.2022586118)
Supplement: Supplementary File [file pnas.2022586118.sapp.pdf]

PNAS
www.pnas.org

Supplementary Information for

The effect of the D614G substitution on the structure of the spike glycoprotein of SARS-CoV-2

Donald J. Benton, Antoni G. Wrobel, Chloë Roustan, Annabel Borg, Pengqi Xu, Stephen R. Martin, Peter B. Rosenthal, John J. Skehel, and Steven J. Gamblin.

Donald J. Benton, Antoni G. Wrobel, John J. Skehel, Steven J. Gamblin.

Email: donald.benton@crick.ac.uk, Antoni.wrobel@crick.ac.uk, john.skehel@crick.ac.uk, steve.gamblin@crick.ac.uk

This PDF file includes:

Figures S1 to S3
SI References

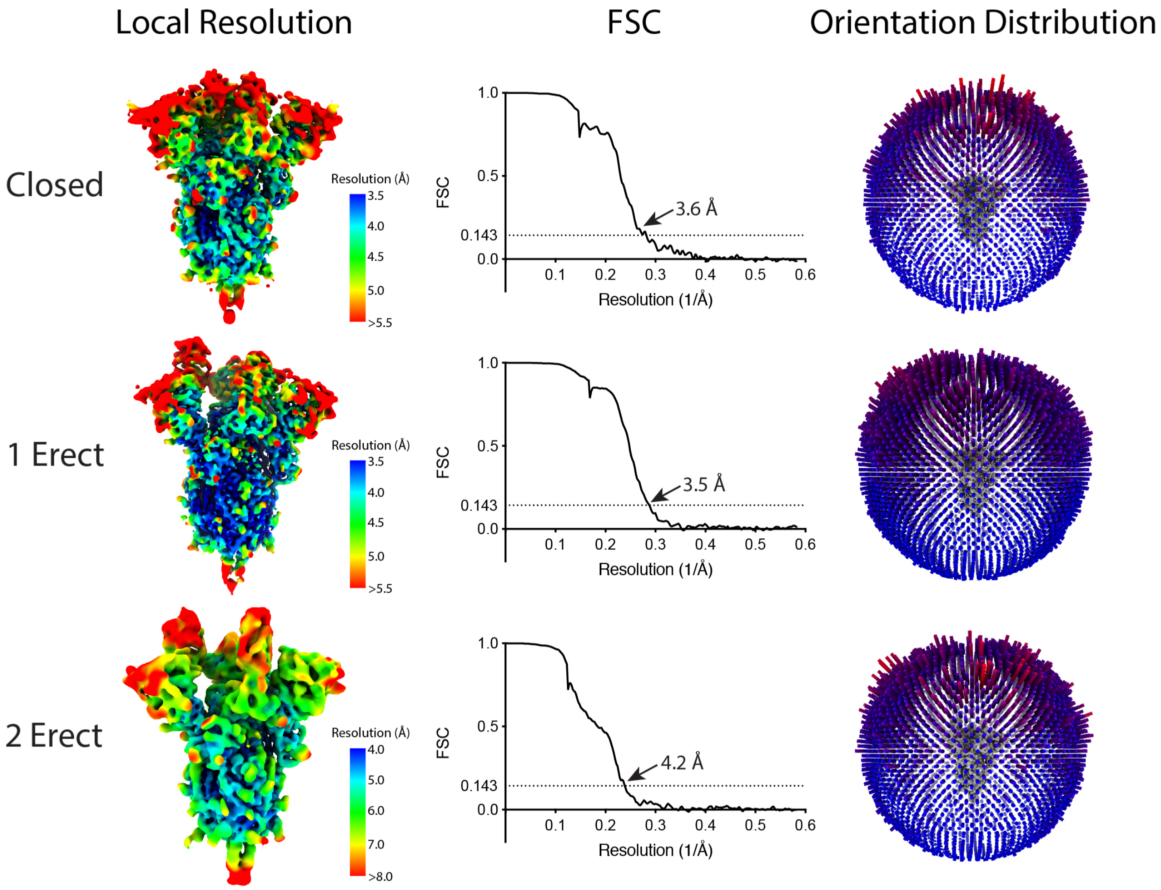

Fig. S1. Maps coloured according to local resolution (left), the corresponding Fourier shell correlation (FSC) plots (middle), and particle orientation distribution plots (right) for the G614 structures determined in this study

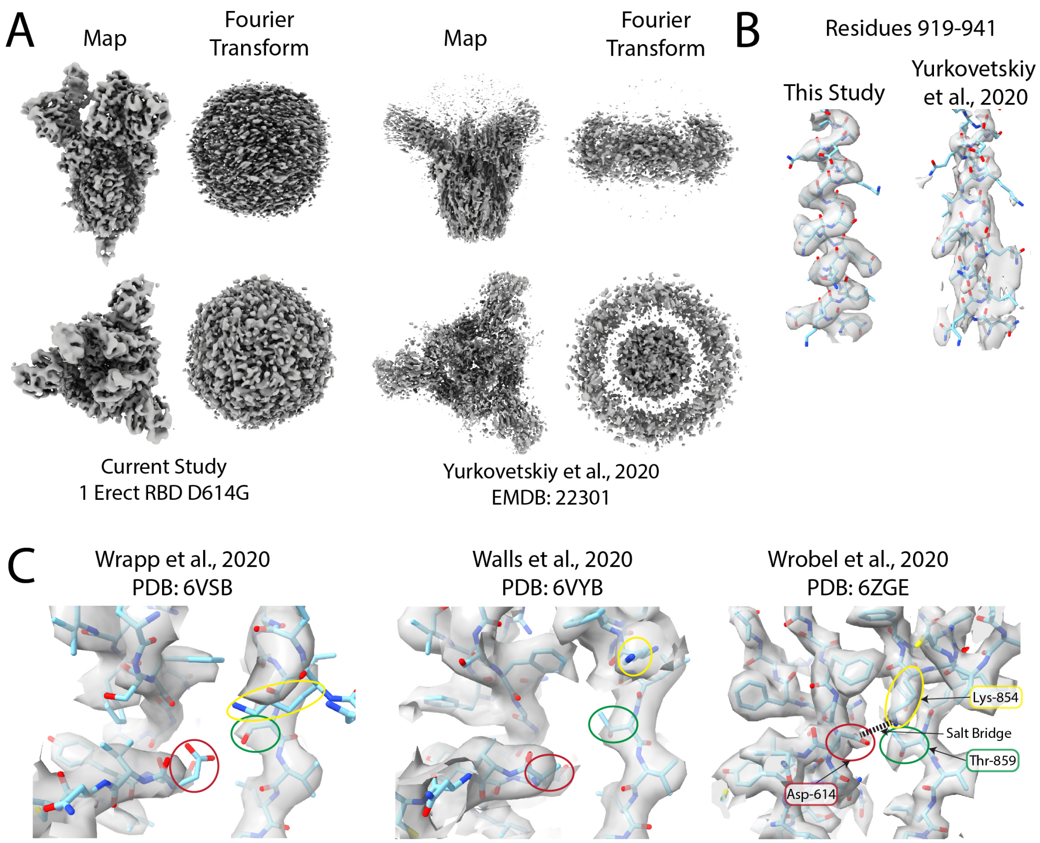

Fig. S2. Density features of maps from this and other studies. (A) Comparison of maps from this current study (left) with those of Yurkovetskiy et al. (1) (right), showing orthogonal views of the real space maps and their Fourier transforms (magnitudes of the complex Fourier components with the constant component set to zero) generated with Chimera (2) on the EMDB-22301 map resampled for comparison to the same size as the maps presented in this study. The map from Yurkovetskiy et al. (right) shows considerable anisotropy in the direction of the symmetry axis, which can be seen in the stretched appearance of the real-space map. This anisotropy can be further observed in the Fourier transform, which resembles a toroid and has substantially more information in a single plane than in the others, likely due to insufficient sampling of the sphere of views. In comparison, the Fourier transform of the map from our current study (left) is a sphere, due to uniform information distribution. The observed pathological anisotropy in the study by Yurkovetskiy et al. is likely a consequence of the limited particle orientation in the collected data, information not provided in their study. (B) The anisotropy in the EMDB-22301 map can be seen to make accurate structural modelling unfeasible, when the density is investigated for a typical S2 helix (919-941). (C) Spike structures from previous studies of D614 spikes, focussing on the regions proximal to residue 614 with models shown in blue and EM density in grey. The residues under question are circled: Asp-614 – red, Lys-854 – yellow, Thr-859 – green. The early studies of SARS-CoV-2 spike (3, 4) (left and middle) have isotropic structures but do not have reliable density in this region consistent with an interaction of residue D614 with neighbouring residues. Our previous structure of the D614 spike (5), which adopts a more closed conformation than earlier studies, indicates a favourable geometry for a salt-bridge interaction between D614 and K854 in the density (right). The higher accuracy of the modelling in our previous study can be seen when comparing per-residue Q-scores (6) of these residues. For residue D614 (PDB:6ZGE) 0.77 compared to 0.41 (PDB:6VSB) and 0.53 [truncated to Ala] (PDB:6VYB). For residue K854 (PDB:6ZGE) 0.79 compared to 0.15 (PDB:6VSB) and 0.61 (PDB:6VYB).Maps coloured according to local resolution (left), the corresponding Fourier shell correlation (FSC) plots (middle), and particle orientation distribution plots (right) for the G614 structures determined in this study

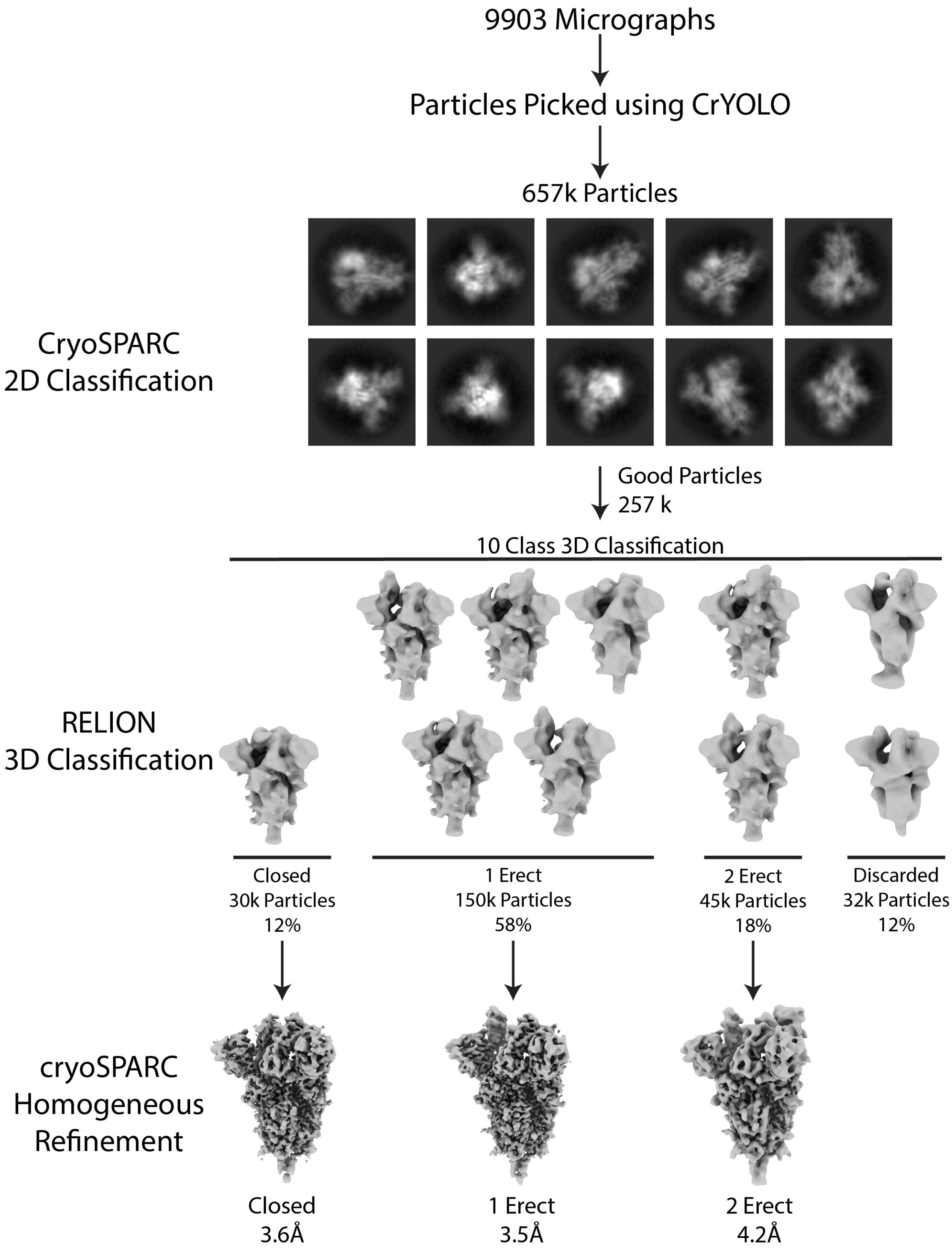

Fig. S3. Image processing scheme for the single particle cryoEM structure determination.

SI References

1. L. Yurkovetskiy, *et al.*, Structural and Functional Analysis of the D614G SARS-CoV-2 Spike Protein Variant. *Cell* (2020) https:/doi.org/10.1016/j.cell.2020.09.032.
2. E. F. Pettersen, *et al.*, UCSF Chimera - A visualization system for exploratory research and analysis. *J. Comput. Chem.* **25**, 1605–1612 (2004).
3. D. Wrapp, *et al.*, Cryo-EM structure of the 2019-nCoV spike in the prefusion conformation. *Science (80-.).* (2020) https:/doi.org/10.1126/science.aax0902.
4. A. C. Walls, *et al.*, Structure, Function, and Antigenicity of the SARS-CoV-2 Spike Glycoprotein (2020) https:/doi.org/10.1016/j.cell.2020.02.058 (May 10, 2020).
5. A. G. Wrobel, *et al.*, SARS-CoV-2 and bat RaTG13 spike glycoprotein structures inform on virus evolution and furin-cleavage effects. *Nat. Struct. Mol. Biol.*, 1–5 (2020).
6. G. Pintilie, *et al.*, Measurement of atom resolvability in cryo-EM maps with Q-scores. *Nat. Methods* (2020) https:/doi.org/10.1038/s41592-020-0731-1.
